# Sprint Acceleration Mechanics in Fatigue Conditions: Compensatory Role of Gluteal Muscles in Horizontal Force Production and Potential Protection of Hamstring Muscles

**DOI:** 10.3389/fphys.2018.01706

**Published:** 2018-11-30

**Authors:** Pascal Edouard, Jurdan Mendiguchia, Johan Lahti, Pierrick J. Arnal, Philippe Gimenez, Pedro Jiménez-Reyes, Matt Brughelli, Pierre Samozino, Jean-Benoit Morin

**Affiliations:** ^1^Inter-University Laboratory of Human Movement Science (LIBM EA 7424), University of Lyon, University Jean Monnet, Saint Etienne, France; ^2^Department of Clinical and Exercise Physiology, Sports Medicine Unity, University Hospital of Saint-Etienne, Faculty of Medicine, Saint-Étienne, France; ^3^Medical Commission, French Athletics Federation, Paris, France; ^4^Department of Physical Therapy, ZENTRUM Rehab and Performance Center, Barañain, Spain; ^5^Université Côte d’Azur, LAMHESS, Nice, France; ^6^Faculty of Sports and Health Sciences, University of Jyväskylä, Jyväskylä, Finland; ^7^Rythm, Research Team, San Francisco, CA, United States; ^8^Laboratory Culture Sport Health Society (EA 4660), University of Bourgogne Franche-Comté, Besançon, France; ^9^Faculty of Sport, Catholic University of San Antonio, Murcia, Spain; ^10^Centre for Sport Studies, King Juan Carlos University, Madrid, Spain; ^11^Sports Performance Research Institute New Zealand, School of Sport and Recreation, Auckland University of Technology, Auckland, New Zealand; ^12^Univ Savoie Mont Blanc, Laboratoire Interuniversitaire de Biologie de la Motricité, EA 7424, Chambéry, France

**Keywords:** hamstring, gluteus maximus, muscle, performance, sprint kinetics, sports injury prevention, risk factors

## Abstract

**Aim:** Hamstring muscle injury is the main injury related to sports requiring sprint acceleration. In addition, hamstring muscles have been reported to play a role in horizontal force production during sprint acceleration performance. The aim of the present study was to analyze (i) the determinants of horizontal force production and (ii) the role of hip extensors, and hamstring muscles in particular, for horizontal force production during repeated sprint-induced fatigue conditions.

**Method:** In this experimental laboratory setting study including 14 sprint-trained male athletes, we analyzed (i) the changes in sprint mechanics, peak torque of the knee and hip extensors and flexors, muscle activity of the *vastus lateralis*, *rectus femoris*, *biceps femoris*, and *gluteus*, and sagittal plane lower limb motion, before and after twelve 6-s sprints separated by 44 s rest on an instrumented motorized treadmill, and (ii) the determinants of horizontal force production (*F_H_*) during the sprint acceleration in a fatigue state (after 12 sprints).

**Results:** The repeated-sprint protocol induced a decrease in maximal power output (*Pmax*) [-17.5 ± 8.9%; effect size (ES): 1.57, large] and in the contact-averaged horizontal force component (*F_H_*) (-8.6 ± 8.4%; ES: 0.86, moderate) but not meaningful changes in the contact-averaged resultant (total) force (*F_Tot_*) (-3.4 ± 2.9%; ES: 0.55, small) and vertical force component (*F_V_*) (-3.1 ± 3.2%; ES: 0.49, small). A decrease was found in concentric peak torque of the knee flexors and extensors and in *gluteus* and *vastus lateralis* muscle activity during entire swing and end-of-swing phase. An increase was found in contact time and swing time, while step frequency and knee speed before ground contact decreased. Muscular determinants associated with *F_H_* and its decrease after the repeated-sprint protocol were concentric peak torque of the hip extensors (*p* = 0.033) and a decrease in *gluteus maximus* activity at the end-of-swing (*p* = 0.007), respectively.

**Conclusion:** Sprint-induced fatigue lead to changes in horizontal force production muscular determinants: hamstring muscle seems not to have the same role than in non-fatigue condition. Horizontal force production seems to be more dependent on the hip extensors and *gluteus maximus* function. Given the fatigue-induced decrease in hamstring muscle strength, we can hypothesize that muscle compensatory and kinematic strategies reported in a fatigued state could be an adaptation to allow/maintain performance and a protective adaptation to limit hamstring muscles constraints.

## Introduction

Hamstring muscle injury (HMI) is, despite improvements in knowledge and prevention strategies, the main injury related to sport requiring sprint acceleration, such as football (Woods et al., 2004; Ekstrand et al., 2011, 2016), rugby (Brooks et al., 2006), and athletics (Opar et al., 2014; Edouard et al., 2016). HMI leads to considerable consequences for athletes, such as time-loss from sport and high risk of recurrence (Woods et al., 2004; Ekstrand et al., 2011, 2016; Malliaropoulos et al., 2011; Edouard et al., 2016). This makes HMI a challenge for all stakeholders around the athletes to better understand and prevent them.

The majority of HMIs occur during sprinting actions, for example for sprinters at or near top speed (Stanton and Purdam, 1989; Askling et al., 2007), or in football winning ball possession, passing a defending player or gaining position to score a goal (Arnason et al., 1996; Woods et al., 2004; Opar et al., 2015). There is a clear link between sprinting activity and HMI occurrence (Stanton and Purdam, 1989; Schache et al., 2012). From this basic standpoint, we suggest that better understanding sprint performance and mechanics is a key parameter to improve HMI prevention.

During the acceleration phase of sprinting, forward orientation of ground reaction force (GRF) has been shown to be a stronger determinant of field sprint performance than the overall magnitude of vertical or resultant GRF (Morin et al., 2011a, 2012; Rabita et al., 2015). Hip extensor muscles (*gluteus maximus* and hamstring muscles) play a key role in this horizontal force production (Dorn et al., 2012; Hamner and Delp, 2013; Morin et al., 2015), and their neuromuscular behavior (strength and EMG) has been linked to an increased risk of sustaining HMI (Croisier et al., 2008; Sugiura et al., 2008; Yeung et al., 2009; Opar et al., 2015; Schuermans et al., 2017). Concretely, a greater amount of horizontal GRF (as averaged over an entire sprint acceleration) was found in subjects who were both able to highly activate their hamstring muscles just before ground contact and had the greatest capacity to produce eccentric knee flexor peak torque (PT) (Morin et al., 2015). In addition to contributing to a net transfer of power from proximal to distal joints, it has been suggested that the bi-articular posterior thigh muscles such as the hamstring muscles have a major influence on controlling the direction of external forces and propel the center of mass in the horizontal/forward direction (Jacobs and van Ingen Schenau, 1992; Jacobs et al., 1996). In addition, in terms of hamstring demands in locomotor tasks, sprinting seems to place the largest demands on this muscle group (van den Tillaar et al., 2017). Coherently, it has been reported that football players returning to play after rehabilitation from an HMI can display a decrease in horizontal force production (Mendiguchia et al., 2014, 2016). Interestingly, *gluteus maximus* activity and peak concentric hip extension torque were also significantly related to the horizontal force production averaged over the initial steps of the acceleration phase (Morin et al., 2015). Thus, from this basic standpoint, it seems logical to expect hip extensors and knee flexors to play a key role in sprinting both from performance and injury prevention perspectives.

In addition to the mechanical and muscular determinants of HMI occurrence in sprinting, another parameter comes into the equation: fatigue. Indeed, injuries do not only occur during the initial sprints. Since team sports matches are associated with muscular fatigue (Silva et al., 2017), and since HMI occurrence significantly increases at the end of each half of football or rugby matches (Woods et al., 2004; Brooks et al., 2006), it is reasonable to assume that HMI risk increases with hamstring muscle fatigue. Mair et al. (1996), in an animal laboratory study, reported that fatigued muscles absorbed less energy before failure compared to unfatigued muscles, suggesting that fatigued muscles may be at higher risk of injury. Therefore, in a HMI prevention perspective, it seems imperative to investigate the influence of fatigue, since it represents what a broad population of athletes experience in practical conditions and is one of the acknowledged HMI risk factors (Opar et al., 2012; Buckthorpe et al., 2018). There is a clear interest to better understand (i) how fatigue influences hamstring muscle function during sprint acceleration, and (ii) what is the role of the hamstring muscles for horizontal force production in fatigue conditions. This could help to better understand and prevent this injury risk associated with sports practice (Buckthorpe et al., 2018; Lord et al., 2018).

In match-induced fatigue conditions (Andersson et al., 2008) or after a simulated match (Rahnama et al., 2003; Greig, 2008; Small et al., 2009, 2010) or after RS (Pinniger et al., 2000; Timmins et al., 2014), changes in sprint kinematics and a decrease in hamstring strength have been reported. These changes could cause higher HMI risk due to: (i) changes in sprint kinematics leading to higher constraints on the hamstring muscles during fatigue (Small et al., 2009; Røksund et al., 2017), and (ii) fatigue-induced decrease in hamstring strength leading to increased HMI risk, as hamstring weakness is an acknowledged modifiable HMI risk factor (Croisier et al., 2008; Yeung et al., 2009; van Dyk et al., 2016). In addition, Morin et al. (2011b) reported that a repeated sprint protocol on an instrumented treadmill induced both a significant decrease in the capability to produce total force (*F_Tot_*) and an even larger relative decrease in horizontal force component (*F_H_*). However, it is unknown whether this decrease in *F_H_* is explained by the decrease in hamstring strength or other mechanisms. To our knowledge, experimental studies exploring the influence of fatigue (Rahnama et al., 2003; Greig, 2008; Small et al., 2009, 2010; Morin et al., 2011b) (i) did not report direct relationships between fatigue and HMIs, and (ii) did not analyze the muscular determinants and the role played by hamstring muscles for horizontal force production in fatigue conditions.

Furthermore, since muscle fatigue is task-specific (Enoka and Duchateau, 2008), it is of interest to analyze hamstring muscles function in sprint-specific fatigue conditions. In addition, in terms of hamstring demands in locomotor tasks, sprinting seems to place the largest demands on this muscle group (van den Tillaar et al., 2017). Thus, a repeated-sprint protocol seems to be a relevant design to analyze both the influence of fatigue on hamstring muscle function during sprint acceleration, and the muscular determinants of horizontal force production in such fatigue conditions. Pinniger et al. (2000) reported that a general hamstring fatigue task (i.e., 10 maximal 40-m sprints separated by 30-s recovery) in addition to a specific hamstring fatigue task on an isokinetic dynamometer lead to a decrease in hamstring PT, as well as changes in sprint kinematics and muscle activity. They interpreted these results as potential evidence for a “protective” mechanism to compensate for the decreased force generation capacity of the fatigued muscles (Pinniger et al., 2000). However, sprint mechanics have not been recorded concomitantly. To our knowledge, no study has presented a global approach recording sprint mechanics, muscle strength and activity after repeated sprint, thus inducing sprint-specific fatigue and a potentially a better scientific understanding of sprint acceleration performance, mechanical determinants, and hamstring muscle function in this context.

Thus, the aim of the present study was to analyze (i) the determinants of the sprint acceleration performance and horizontal force production and (ii) the role of hip extensors, and hamstring muscles in particular, for the horizontal force production, in repeated sprint-induced fatigue conditions.

## Materials and Methods

### Study Design

This was an experimental laboratory setting, cross-sectional study analysing sprint mechanics, isokinetic PT of knee and hip extensors and flexors, muscle activity and sagittal plane lower limb motion, before and after twelve 6-s sprints on an instrumented motorized treadmill. The study was approved by the institutional ethics review board of the Faculty of Sport Sciences, and conducted according to the Declaration of Helsinki II.

### Participants

Fourteen male subjects (body mass (mean ± SD): 79.9 ± 7.9 kg; height 1.79 ± 0.07 m; age 24.2 ± 4.6 years) trained for sprint running (seven football and basketball competitive level players, four under-23 high-level rugby union players, and three regional to national-level track and field athletes) volunteered to participate in this study. All subjects trained at least three times a week since more than 3 years, and were free of musculoskeletal pain or injuries at the time of the study and in the six previous months. Written informed consent was obtained from the subjects.

### Experimental Protocol and Repeated Sprints Protocol

A familiarization session for treadmill sprints and isokinetic tests was performed approximately 1 week prior to the testing session. After a standardized warm-up described below, subjects repeated short (<5 s) treadmill sprints at increasing intensities, with full recovery and until being comfortable with the running technique required (∼6 trials). Following this, the subjects performed a familiarization session with the isokinetic test procedure for the knee flexors (KFlex) and extensors (KExt) and hip flexors (HFlex) and extensors (HExt), following the isokinetic testing procedure described below, during which they were encouraged to perform at their best.

For the testing session, the standardized warm-up consisted of 5 min of 10 km.h^-1^ running, followed by 5 min of sprint-specific hamstring warm-up exercises, and three progressive 6-s sprints at increasing velocities separated by 2 min of passive rest. Subjects performed the isokinetic warm-up followed by maximal isokinetic strength measurements of KFlex, KExt, HFlex, and HExt. Thereupon, EMG electrodes and reflective markers were placed on the right lower limb. Maximal EMG activity was measured for each muscle group for standardization. Subjects repeated the sprint specific warm-up on the treadmill with two submaximal 6-s sprints. After 5 min of recovery, the subjects performed one maximal 6-s sprint, from which maximal power output (*Pmax*) was used as the criterion score for the first sprint of the RS performed during the testing session. Indeed, to prevent pacing effects occurring in such RS protocols (Billaut et al., 2011), subjects were requested to achieve at least 95% of their respective criterion score during the first sprint of the RS testing session (Morin et al., 2011b). Subjects were then allowed ∼3 min of free cool-down prior to the RS protocol. The RS consisted in performing twelve 6-s sprints separated by 44-s of passive rest. Subjects exercised to protocol completion or volitional fatigue, whichever occurred first. Sprint mechanical data, EMG activity and video data were recorded during each sprint, and before and 3 min after RS maximal isokinetic strength of KFlex, KExt, HFlex, and HExt were recorded.

### Isokinetic Testing Procedure

Isokinetic strength was measured using a Con-Trex^®^ isokinetic dynamometer (Con-Trex MJ; CMV AG, Dübendorf, Switzerland), following the same standardized procedure after instructions and conducted by the same examiner (PE). PT of KFlex, KExt, HFlex, and HExt was obtained during three maximal repetitions at 120°/s, in concentric and eccentric mode in a randomized order (Morin et al., 2015). Only the right lower limb was tested. Gravity corrections were incorporated and artifacts were controlled (Maffiuletti et al., 2007; Julia et al., 2010). Subjects were given oral encouragement without visual feedback. Before maximal measurement and only before RS, each subject performed two series of six graded submaximal concentric repetitions at 120°/s, followed by three submaximal repetitions at 120°/s in the concentric and eccentric mode in a random order, as a specific isokinetic warm-up. This angular velocity (120°/s) was chosen because (i) it was previously used in experimental studies on fatigue-tasks (Rahnama et al., 2003; Small et al., 2009), (ii) showed a high inter-session reliability (Maffiuletti et al., 2007), and (iii) we wanted to set only one velocity to avoid the bias induced by multiple isokinetic series. A 60-s rest separated each series of movements (Morin et al., 2015). For KFlex and KExt measurements, each subject was seated on the dynamometer, with 105° of hip flexion, with auto adhesive straps placed across the chest and pelvis, support to stabilize the contralateral limb, and with instruction to grip the seat during maximal measurements (Morin et al., 2015). The knee rotational axis was aligned with the dynamometer rotational axis. The dynamometer shin pad was attached 2–3 cm proximal to the malleoli. The range of knee motion was fixed at 90° (from full extension to 90° of knee flexion) (Morin et al., 2015). For HFlex and HExt measurements, each subject laid in the supine position, with the pelvis and chest stabilized by auto adhesive straps, the hip in the sagittal plane and the knee flexed at 90° (Julia et al., 2010; Morin et al., 2015). The contralateral leg rested on a support under the foot, with 0° of hip extension and 90° of knee flexion (Julia et al., 2010; Morin et al., 2015). The dynamometer rotational axis was aligned with the trochanter major, and the tested side was attached to the dynamometer via a thigh strap. The range of hip motion was fixed at 90° (from 10° of hip extension to 80° of flexion) (Morin et al., 2015). PT normalized to body weight (PTBW, in Nm.kg^-1^) and agonist-to-antagonist ratios were used. Reliability of each parameter was calculated using maximal data from the familiarization and testing session (Hopkins, 2000; Maffiuletti et al., 2007): reliability for KFlex and KExt was high [for PT: intraclass correlation coefficient (ICC): 0.86–0.95; SEM: 3.8–8.5%; and CV: 3.0–5.7%; for ratios: ICC: 0.69–0.85; SEM: 6.2–7.5%; and CV: 5.5–5.7%]; reliability for HFlex and HExt was moderate (for PT_BW_: ICC: 0.60–0.78; SEM: 9.6–19.4%; and CV: 8.0–17.3%; for ratios: ICC: 0.20–0.55, SEM: 10.6–21.3%; and CV: 7.0–19.6%).

### Sprint Performance Variables

Sprint mechanics were measured during sprints performed on a motorized instrumented treadmill (ADAL3D-WR, Medical Development – HEF Tecmachine, Andrézieux-Bouthéon, France) (for full details, see Morin et al., 2010). Subjects started in a typical crouched sprint-start position with their preferred foot forward, attached with a leather weightlifting belt and thin stiff rope to the wall behind. According to previous studies (Morin et al., 2010, 2011a, 2012, 2015), sprint kinematics [contact time (*t_c_* in s), aerial time (*t_a_* in s), swing time (*t_swing_* in s) and *SF* (Hz)] and sprint kinetics [contact-averaged horizontal (*F_H_*, BW) and vertical (*F_V_*, BW) force component, resultant (total) force (*F_Tot_*, BW), maximal velocity (*V_max_*, m.s^-1^), and maximal power output (*P_max_*, W.kg^-1^) were calculated using all steps from start until *V_max_*].

### Muscular Activity

**FIGURE 1:**
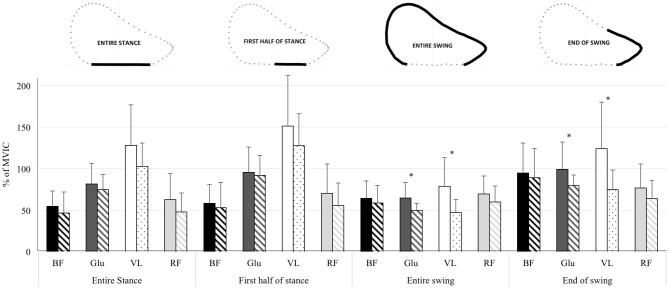
Mean ± standard deviation EMG activity [represented by the percentage of the maximal voluntary isometric contraction (MVIC)] of the *biceps femoris* (BF), *gluteus maximus* (Glu), *vastus lateralis* (VL) and *rectus femoris* (RF) muscles over the entire sprint acceleration (all steps), for the different phases of the step cycle, and for the first sprints (Plain histograms) and last sprints (scratch histograms) of the repeated-sprint protocol values. ^∗^ significant differences (*p*-value < 0.05) and at least moderate effects (effect size > 0.6) between first and last sprints values.

EMG activity of the right *vastus lateralis* (VL), *rectus femoris* (RF), BF and *gluteus maximus* (Glut) muscles was recorded using bipolar silver chloride surface electrodes of 30 mm diameter (Meditrace 100, Tyco healthcare, Mansfield, ON, Canada) placed on the skin according to recommendations by SENIAM (Hermens et al., 2000), with low impedance (Z < 5 kΩ) at the skin-electrode surface, and with the reference electrode on the patella. EMG data were recorded with PowerLab system (16/30 – ML880/P, ADInstruments, Bella Vista, NSW, Australia) with a sampling frequency of 2,000 Hz. The EMG signal was amplified with octal bio-amplifier (Octal Bioamp, ML138, ADInstruments) with a bandwidth frequency ranging from 5 to 1,000 Hz (input impedance = 200 MΩ, common mode rejection ratio = 85 dB), transmitted to the computer and analyzed with LabChart 7.3 software (ADInstruments). Vertical GRF and EMG signals for the right leg were time synchronized on LabChart 7.3. EMG activity of each muscle was quantified using the root mean square (RMS) with a 20-ms moving window, and recorded during the following phases of the running cycle for the right leg: (i) first half of the stance phase, (ii) entire stance phase as detected by a 30-N threshold, (iii) entire swing phase (from foot takeoff to the subsequent landing of the same foot), and (iv) end-of-swing phase, defined as the aerial phase (no foot-ground contact) preceding the stance phase (Figure 1). RMS data for all phases were normalized to maximal voluntary isometric contractions (MVIC) data obtained during two 3-s efforts. MVICs were performed in the sagittal plane to assess hip extension (Glut) and knee flexion (BF) and extension (VL and RF) for the right hip and knee with a constant angle. Hip extension was tested with subjects laying on a table in a prone position, 30° hip flexion and the knee fully extended. Knee extension and flexion were tested with subjects seated in the frame of a Cybex II (Ronkonkoma, NY, United States) seat, fastened to the frame at the pelvis and with knee and hip angles set at 90°. During these three sets of two MVICs, two experimenters applied manual resistance at the subjects’ ankles to ensure a safe maximal isometric exertion.

### Sagittal Plane Lower Limb Motion

The motion of the right foot and knee was recorded in the sagittal plane of motion with a camera (sampling rate of 120 frames per second, Basler scA640-120gc, Basler AG, Germany) mounted on a tripod placed 1.5 m away from the treadmill in a lateral view (for more information, see Morin et al., 2015). Retro-reflective markers were placed onto the great trochanter, the lateral femoral epicondyle, and the fifth metatarsal head. Marker trajectories in the sagittal plane (vertical and horizontal directions) were tracked and analyzed with Simi Motion 2D software (Simi Reality Motion Systems GmbH, Unterschleissheim, Germany). The kinematic variables of interest were: foot and knee speed before contact (in m.s^-1^) and foot acceleration before ground contact (in m.s^-2^).

### Data Analysis and Statistics

Descriptive statistics are presented as mean values ± SD. Normal distribution of the data was checked by the Shapiro–Wilk normality test. For all sprint mechanics and EMG variables considered, “first sprints” and “last sprints” values were averaged for the first two and last two sprints for each subject, respectively (Morin et al., 2011b). For isokinetic values, we considered measurements performed before RS as “pre-RS” and after RS as “post-RS.” The significance level was set at *P* < 0.05. Analyses were performed using Excel (Office, Microsoft^®^, 2017) and JASP (JASP Team software, Version 0.8.5.1, University of Amsterdam, Netherlands).

In order to estimate the influence of the RS protocol, we compared first vs. last sprints sprint mechanics and muscle activity values, pre-RS vs. post-RS muscle PT and ratio values, using paired samples *t*-tests and standardized differences in means for practical significance with ES threshold values of <0.2, 0.2, 0.6, 1.2, 2.0, and 4.0 to represent trivial, small, moderate, large, very large and extremely large effects, respectively (Hopkins et al., 2009). Only moderate or higher effects were kept for interpretation.

In order to analyze the determinants of the horizontal force production (*F_H_*, dependent variable) we used a stepwise multiple regression model including last sprints and post-RS values and percentage of difference between the first and last sprints muscular activity values and between pre- and post-RS muscular PT and ratio values (delta-RS) as independent variables.

## Results

### Comparison of Values Before and After the Repeated-Sprint Protocol

The comparison of first vs. last sprints and pre-RS vs. post-RS values is presented in Table 1, and for muscle activity according to the different phases of the step cycle in Figure 1. The RS protocol induced a large decrease in sprint acceleration performance: mean decrease in *P_max_* was -17.5 ± 8.9% (ES: 1.57, large). There was also a decrease in *V_max_* (-6.6 ± 6.7%; ES: 0.86, moderate) and in *F_H_* (-8.6 ± 8.4%; ES: 0.86, moderate), but not meaningful in *F_Tot_* (-3.4 ± 2.9%; ES: 0.55, small) and in *F_V_* (-3.1 ± 3.2%; ES: 0.49, small). There were moderate to large changes in step temporal and kinematics variables with the RS protocol: increase in contact time (11.8 ± 9.8%; ES: 1.30, large) and swing time (5.2 ± 9.1%; ES: 0.77, moderate), decrease in *SF* (-6.0 ± 7.7%; ES: 0.91, moderate), decrease in knee speed before ground contact (-14.1 ± 11.0%; ES: 1.56, large). For muscular aspects, we observed a moderate decrease in KFlex (-11.9 ± 13.1%; ES: 0.70, moderate) and KExt (-5.8 ± 8.8%; ES: 0.73, moderate) concentric PT, and in *gluteus maximus* muscle activity during entire swing (-19.9 ± 16.4%; ES: 0.81, moderate) and end-of-swing (-15.5 ± 16.3%; ES: 0.60, moderate) phases and in *vastus lateralis* muscle activity during entire swing (-33.6 ± 21.9%; ES: 0.92, moderate) and end-of-swing (-32.3 ± 23.8%; ES: 0.88, moderate) phases.

**Table 1:** Comparison of values between first sprints/before pre-repeated sprints (Pre-RS) and last sprints/after repeated sprints (post-RS) using *t*-test for paired samples and effect size [with 95% confident interval (95% CI)] for practical significance.

Parameters	First sprints or Pre-RS values	Last sprints or Post-RS values	*p*-values	Percentage of changes	Effect size (upper-lower 95% CI)	
*Sprint performance variables*						
***P_max_* (W.kg^-1^)**	**22.2 (2.5)**	**18.3 (2.5)**	**0.000**	**-17.5 (8.9)**	**1.57 (1.08–2.07)**	**Large**
***F_H_* (BW)**	**0.351 (0.036)**	**0.320 (0.038)**	**0.003**	**-8.6 (8.4)**	**0.86 (0.36–1.36)**	**Moderate**
*F_V_* (BW)	1.62 (0.10)	1.57 (0.09)	0.002	-3.1 (3.2)	0.49 (0.22–0.80)	Small
*F_Tot_* (BW)	1.66 (0.10)	1.61 (0.09)	0.001	-3.4 (2.9)	0.55 (0.30–0.84)	Small
***V_max_* (m.s^-1^)**	**6.61 (0.53)**	**6.15 (0.44)**	**0.002**	**-6.6 (6.7)**	**0.86 (0.37–1.35)**	**Moderate**
*Step temporal variables*						
**Contact time (s)**	**0.152 (0.013)**	**0.169 (0.016)**	**0.000**	**11.8 (9.8)**	**1.30 (0.77–1.92)**	**Large**
Aerial time (s)	0.094 (0.009)	0.094 (0.011)	0.937	0.1 (10.9)	0.02 (-0.56–0.67)	Negligible
**Swing time (s)**	**0.338 (0.021)**	**0.355 (0.026)**	**0.048**	**5.2 (9.1)**	**0.77 (0.00–1.57)**	**Moderate**
**Step frequency (Hz)**	**4.091 (0.280)**	**3.840 (0.280)**	**0.014**	**-6.0 (7.7)**	**0.91 (0.22–1.61)**	**Moderate**
*Motion analysis*						
Foot speed before contact (m.s^-1^)	-5.40 (0.60)	-5.16 (0.60)	0.003	-4.35 (4.20)	0.39 (0.17–0.62)	Small
**Knee speed before contact (m.s^-1^)**	**-3.41 (0.31)**	**-2.93 (0.46)**	**0.001**	**-14.1 (11.0)**	**1.56 (0.79–2.30)**	**Large**
Foot acceleration before contact (m.s^-2^)	-95.8 (15.1)	-88.8 (14.3)	0.002	-7.2 (6.3)	0.47 (0.21–0.72)	Small
*Isokinetic variables (Nm.kg^-1^)*						
**KFlex con**	**1.73 (0.34)**	**1.49 (0.20)**	**0.006**	**-11.9 (13.1)**	**0.70 (0.23–1.16)**	**Moderate**
KFlex ecc	2.29 (0.47)	2.11 (0.42)	0.04	-7.3 (11.8)	0.38 (0.02–0.76)	Small
**KExt con**	**2.67 (0.22)**	**2.51 (0.26)**	**0.024**	**-5.8 (8.8)**	**0.73 (0.11–1.35)**	**Moderate**
KExt ecc	3.92 (0.58)	3.60 (0.66)	0.002	-8.3 (7.9)	0.55 (0.24–0.85)	Small
Ratio KFlexcon/KExtcon	0.65 (0.12)	0.60 (0.08)	0.01	-6.7 (9.5)	0.42 (0.11–0.73)	Small
Ratio KFlexecc/KExtcon	0.86 (0.16)	0.85 (0.19)	0.75	-1.1 (13.7)	0.06 (-0.34–0.49)	Negligible
HExt con	2.56 (0.42)	2.32 (0.36)	0.002	-8.9 (7.9)	0.57 (0.25–0.89)	Small
HExt ecc	3.36 (1.08)	3.37 (1.04)	0.955	1.7 (15.4)	0.01 (-0.24–0.23)	Negligible
HFlex con	2.28 (0.33)	2.17 (0.31)	0.076	-4.5 (9.6)	0.34 (-0.04–0.72)	Small
HFlex ecc	2.97 (0.79)	2.68 (0.65)	0.02	-8.5 (13.9)	0.37 (0.06–0.69)	Small
Ratio Extcon/Flexcon	1.13 (0.16)	1.08 (0.20)	0.373	-3.3 (15.9)	0.28 (-0.38–0.93)	Small
Ratio Extecc/Flexcon	1.46 (0.37)	1.54 (0.36)	0.228	7.3 (18.3)	0.21 (-0.15–0.57)	Small
*Muscle activity (% of MVIC)*						
Entire stance BF	54.7 (18.3)	46.6 (25.4)	0.028	-19.5 (21.7)	0.44 (0.06–0.82)	Small
Entire stance glut	81.4 (25.0)	74.8 (18.5)	0.05	-5.7 (13.8)	0.26 (0.00–0.53)	Small
Entire stance VL	127.9 (49.1)	102.9 (28.0)	0.083	-14.1 (21.7)	0.51 (-0.08–1.09)	Small
Entire stance RF	62.9 (31.3)	48.0 (22.8)	0.001	-22.4 (16.7)	0.47 (0.24–0.71)	Small
First half of stance BF	58.4 (22.7)	53.1 (30.5)	0.192	-14.2 (21.8)	0.23 (-0.13–0.60)	Small
First half of stance glut	95.6 (30.4)	92.0 (24.0)	0.306	-1.3 (13.3)	0.12 (-0.12–0.35)	Negligible
First half of stance VL	151.3 (61.3)	127.6 (38.4)	0.171	-10.1 (22.8)	0.39 (-0.19–0.96)	Small
*Muscle Activity (% of MVIC)*				
First half of stance RF	70.4 (35.4)	55.7 (26.9)	0.001	-20.2 (16.0)	0.41 (0.20–0.63)	Small
Entire swing BF	64.3 (20.7)	58.8 (21.1)	0.034	-8.1 (13.5)	0.26 (0.02–0.50)	Small
**Entire swing glut**	**64.7 (18.5)**	**49.8 (8.7)**	**0.006**	**-19.9 (16.4)**	**0.81 (0.28**–**1.33)**	**Moderate**
**Entire swing VL**	**78.9 (34.3)**	**47.2 (15.6)**	**0.006**	**-33.6 (21.9)**	**0.92 (0.31**–**1.52)**	**Moderate**
Entire swing RF	69.5 (21.7)	60.1 (19.0)	0.004	-12.4 (13.5)	0.44 (0.17–0.70)	Small
End of swing BF	95.0 (35.8)	89.1 (35.5)	0.085	-5.6 (11.8)	0.16 (-0.03 – 0.36)	Negligible
**End of swing glut**	**99.2 (32.9)**	**79.6 (13.0)**	**0.024**	**-15.5 (16.3)**	**0.60 (0.09**–**1.10)**	**Moderate**
**End of swing VL**	**124.1 (56.1)**	**74.6 (23.8)**	**0.01**	**-32.3 (23.8)**	**0.88 (0.25**–**1.51)**	**Moderate**
End of swing RF	76.7 (29.1)	64.1 (21.7)	0.007	-14.0 (15.2)	0.43 (0.14–0.73)	Small


*Values are presented with mean (standard deviation). Significant differences (p < 0.05) with moderate or more effects were highlight in bold.P_max_, maximal power output; F_H_, horizontal ground force; F_v_, vertical ground force; F_Tot_, total ground force; V_max_, maximal velocity; KFlex, knee flexors; KExt, knee extensors; HFlex, hip flexors; HExt, hip extensors; ecc, eccentric mode of contraction; con, concentric mode of contraction; BF, biceps femoris; Glu, gluteus maximus; VL, vastus lateralis; RF, rectus femoris; MVIC, maximal voluntary isometric contraction.*

### Determinants of Horizontal Force Production After Repeated Sprints

When explaining last sprints horizontal force production with stepwise multiple regression model using last sprints and post-RS values, the muscular determinant associated with *F_H_* after RS (i.e., in a fatigue state) was post-RS HExt concentric PT (*p* = 0.033). When explaining the changes in horizontal force production between first and last sprints with stepwise multiple regression model using delta-RS values, the muscular determinant associated with the decrease in *F_H_* was the decrease in *gluteus maximus* activity at the end-of-swing (*p* = 0.007).

## Discussion

The main findings of the present study were that (i) the RS protocol induced a clear decrease in sprint acceleration performance (*P_max_*) and in horizontal force production (*F_H_*), which can be considered markers of neuromuscular fatigue, (ii) higher *F_H_* in a fatigue state was mainly associated with a higher concentric PT of the hip extensors, (iii) the lower decrease in horizontal force production after fatigue was mainly associated with a lower decrease in *gluteus maximus* activity at the end-of-swing, and (iv) hamstring muscle torque during knee flexion was not associated with horizontal force production in a fatigue state, contrary to what was observed in the non-fatigue condition (Morin et al., 2015).

### Determinants of Sprint Acceleration Performance in Fatigue Conditions

Our results showed a decrease in *P_max_* in the fatigue condition. This could be caused by a fatigue-induced decrease (i) in the total amount of force produce by the athletes onto the ground (decrease in *F_Tot_*), or (ii) in the ability to orient the force in the horizontal direction (represented by *F_H_* in the present study), or (iii) both. Since *F_H_* decreased with RS while *F_Tot_* was not meaningfully affected, it is very likely that the decrease in *P_max_* in the fatigue condition was mainly explained by the decrease in force output in the horizontal direction (*F_H_*) rather than the total amount of force produced. This is in agreement with a previous study using repeated sprint-induced fatigue (Morin et al., 2011b), and with previous findings in non-fatigue condition reporting that the ability to produce and apply high levels of *F_H_* over the entire sprint acceleration represents a strong determinant of field sprint performance (e.g., Morin et al., 2011a, 2012; Rabita et al., 2015). Thus, whatever the fatigue state, sprint acceleration requires high amounts of force applied in the horizontal direction. This reinforces the interest of a performance-focused perspective to analyze the muscular determinants of the horizontal force production in fatigue conditions.

### Muscular Determinants of Horizontal Force Production in Fatigue Conditions

In non-fatigue conditions, hamstring muscles have been reported to play a key role in horizontal force production (Morin et al., 2015). In a fatigue state, in this population of athletes, the main muscular factor associated with *F_H_* production was hip extensor concentric PT assessed by isokinetic dynamometer within the 3 min after the RS. In addition, the lower decrease in *F_H_* was associated with a lower decrease in *gluteus maximus* activity during the end-of-swing. Hamstring muscles do not seem to have the same association with *F_H_* than in non-fatigue condition. A possible interpretation is that hip extensors (and mainly *gluteus maximus*) play a relatively more important role in horizontal force production in fatigue condition. Hip extensor function was associated with non-fatigued *F_H_* production in the initial part of the acceleration (first 10 steps) (Morin et al., 2015). Moreover, Schache et al. (2014) reported that progressing running speed from jogging to sprinting was mostly dependent on ankle and hip muscle performance. Everything happens as if, in fatigued state, the hip extensors maintained their primary role throughout the acceleration. We hypothesize that muscles playing a more important role in the second part of the acceleration (and thus over the entire acceleration) in non-fatigue condition (i.e., hamstring muscles) (Morin et al., 2015) do not equally assume this role in an fatigued condition. Hip extensors (i.e., *gluteus maximus*) may then compensate the potentially altered hamstring muscle function in a synergistic manner.

Further support to this interpretation was the observed decrease in knee flexor concentric PT in fatigue condition, which could lead to consequences for muscle action management to allow *F_H_* production. Indeed, due to muscle redundancy various neuro-motor strategies may exist to compensate for decreased muscle strength (Goldberg and Neptune, 2007). The muscular pattern could shift with fatigue to maintain a forward-oriented force production given the greater decrease in knee flexors compared hip extensors PT (Table 1). This synergist hypothesis is consistent with walking studies showing that *gluteus maximus* and hamstring muscles compensated for one another (Jonkers et al., 2003; Komura and Nagano, 2004; Goldberg and Neptune, 2007): when gluteal muscle strength was reduced, positive work from hamstring muscles increased; when hamstring muscles strength was decreased, the primary compensatory strategy was an increased work output from *gluteus maximus*.

In the sprint acceleration context, compensatory strategies could be an adaptation to maintain performance. It could also be interpreted as a protective adaptation to limit hamstring muscles constraints and risk of damage. Indeed, it has been suggested that a decreased ability of muscle to generate force is thought to reduce energy absorption efficiency of the muscle which, in turn, can increase potential for musculotendinous injuries (Garrett, 1990; Mair et al., 1996). As a result of the weaker state of the hamstring muscles/knee flexors with fatigue, the hamstring muscles might be further exposed to injury risk, especially when high levels of force, velocity and power production are needed to produce maximal sprint acceleration. We suggest that muscle compensatory strategies observed in the present study, with more important role of *gluteus maximus* in sprint acceleration, are a possible mechanism to maintain performance and protect hamstring muscle in fatigue condition.

In addition, the changes observed in sprint kinematics could also be associated with hamstring muscles protection. We reported a decrease in *SF*, in agreement with Dal Pupo et al. (2017), as an adaptation of sprint kinematic to the fatigue. We also reported changes in step temporal variables and decreased knee speed before the ground contact (Table 1). These results would be in relation with the reduced maximum combined hip flexion and knee extension angle reported by Small et al. (2009) after simulated football match in combination with the decreased leg angular velocity reported immediately before foot ground contact after fatigue showed by Pinniger et al. (2000). Both authors interpreted these findings as a potential protective mechanism to reduce the rapid lengthening of the hamstring muscles during fatigued sprint running. In agreement with this hypothesis, but contrary to our present results, Dal Pupo et al. (2017) reported an increase of the leg angular velocity in fatigue conditions, and suggested that could be due to the hamstring muscles strength decrease leading to difficulties to decelerate the knee extension, and exposing the hamstring muscles to higher injury risk in fatigue conditions. The changes in kinematics variables reported by Small et al. (2009) and Pinniger et al. (2000) have also been interpreted as limiting the “pawing action” of the lower limb prior to ground contact considered by different authors (Mann and Sprague, 1980; Wiemann and Tidow, 1995) as the most likely functional possibility to produce high amounts of *F_H_*.

### Explanatory Hypothesis for Hamstring Injury Risk in Fatigue Condition

Our findings and our hypothesis on compensatory strategies could help to better understand/explain why a high risk of HMI has been reported in a fatigue context (Woods et al., 2004; Brooks et al., 2006). Fatigue-related weakness of the hamstring muscles should be compensated by hip extensors/*gluteus maximus* to allow horizontal force production and protect the hamstring muscles, as we hypothesized. However, in case the hip extensors/*gluteus maximus* muscles strength is impaired, due to pre-fatigue or fatigue-induced weakness, they cannot perform these roles. This scenario would place the hamstring muscles in a high-demand context, possibly higher than they can assume because of their fatigue state, and consequently expose them to increased potential damage. This hypothesis is further supported by the results of previous studies reporting the role of hip extensors/*gluteus maximus* as HMI risk factor (Sugiura et al., 2008; Schuermans et al., 2017). Weaker concentric hip extensors and eccentric knee flexors PT have been associated with higher risk of HMI occurrence (Sugiura et al., 2008). Schuermans et al. (2017) also reported that lower amounts of *gluteus maximus* activity during the front swing phase of sprint was associated with higher risk of HMI occurrence in football players. Time-dependent muscle activity analysis revealed that players appear to be relatively protected against HMIs when the proximal muscles are recruited to a greater extent throughout the swing phase of sprinting. They suggested that it is very plausible that the hamstrings might be exposed to higher mechanical loading and have to engage in higher metabolic output when the supporting proximal musculature does not function in time (Schuermans et al., 2017). This is also in agreement with Thelen et al. (2006) suggesting that the lumbo-pelvic region muscles affect HMI risk more than that for the distal muscles of the knee and ankle.

### Perspectives for Hamstring Injury Prevention

Following our hypothesis, we suggest that HMI prevention strategies should include hamstring muscle strengthening in both knee flexors and hip extensors function, as well as *gluteus maximus* strengthening as hip extensors, in agreement with Sugiura et al. (2008).

In addition, our present findings support the interest of analysing fatigue-related aspects when managing athletes in the context of HMI primary and secondary prevention. This is in agreement with previous studies analysing hamstring muscle function in fatigue conditions in athletes with history of HMI (Røksund et al., 2017; Lord et al., 2018). Lord et al. (2018) reported a decrease in the knee flexors PT after fatiguing exercises (isokinetic or RS) on the limb with previous HMI compared to contralateral healthy limb or control group. Røksund et al. (2017) reported a significant decrease in running speed with fatigue in football players reporting previous HMIs compared to uninjured players. They suggested that the higher drop in speed during the repeated sprint testing may be an indication of increased disposition to hamstring muscle fatigue in players with previous HMI and concluded about the need for targeted reconditioning programs to ensure complete post-injury rehabilitation in players recovering from HMI (Røksund et al., 2017). Therefore, keeping in mind that HMIs do not only occur during the initial sprint of a match or a training session (Pinto et al., 2018), we think that it is relevant to also perform tests/evaluations investigating hamstring muscle in fatigue conditions. This is in agreement with Mendiguchia et al. (2017) reporting the interest of global rehabilitation program after HMI, where single leg bridge test (Freckleton et al., 2014) for endurance was included as a criterion to meet before clearance for returning to sports in secondary prevention. In addition, Van Der Horst et al. (2017) discussed the interest of evaluating the repeated sprint ability to help determine the return to sport after HMI. We can also hypothesize that the reduced ability to continue maintaining performance when repeating sprints could be an indirect parameter to detect athletes with higher HMI risk, since such decrease in sprint acceleration performance in a fatigue state would mean decrease horizontal force production, as well as hip extensors/*gluteus maximus* performance, which could place the hamstring muscles at risk.

### Perspectives for Sprint Training

Our findings support the interest of compensatory muscle and kinematic strategies to help sprinting performance despite repetitions leading to fatigue. In a performance perspective, we can suggest adding an endurance element in strengthening for lower limb, mainly for posterior chain muscles since they are involved in sprint acceleration performance. Such an approach seems relevant since a short-term conditioning program (4 weeks) with either a maximum strength or a muscular endurance emphasis can equally reduce fatigue-induced loss of strength over a football match (Matthews et al., 2017). In addition, we suggest improving sprint technical effectiveness, and also as specific strengthening exercises for muscles implicated in sprint mechanics (Morin et al., 2017; van den Tillaar et al., 2017) during fatigue conditions. All these strategies could also help in an overall win-win (performance-prevention) strategy.

### Methodological Considerations

The main strengths of the present study were the experimental design and materials used. The motorized instrumented treadmill used allowed performing realistic sprint accelerations from zero to almost maximal velocity in kinetic conditions that are subjectively and objectively comparable to field linear sprinting (Morin et al., 2010; Morin and Sève, 2011). It also allowed measuring sprint kinetics for all the steps, and synchronizing other analyses such as kinematics and EMG (Morin et al., 2015). Such an experimental context represents a great opportunity to improve knowledge on sprint mechanics, and consequently sprint-related issues such as injury risk factors. In addition, this specific setup allowed performing a fatigue protocol using the specific task of sprint accelerations, while continually recording sprint mechanics and muscular activity.

Some limitations related to the methodology used have previously been discussed (Morin et al., 2011b, 2015). Other limitations, such as the small number of subjects, limit the generalization of the findings. However, the small number of subjects is reflective of an ethical exclusion criteria regarding injury history. The heterogeneity of sports participation allows application of the present results to the most frequent sports involving sprint acceleration (football, rugby, basketball and track and field). Future research might consider both sex, age and sports-specificity effects in different level of population including elites. In this study, we only quantified isokinetic PT value, and further research might be directed toward the angle at which PT is attained and also the area under the torque curve time history (total work). Assessment of HFlex and HExt has been performed in the supine position, which is possibly not be the most optimal position, since Guskiewicz et al. (1993) suggested that anatomical, physiological, and biomechanical factors should be examined while standing, because the prone or supine position does not permit optimal torque generation. Only muscle activity the long head of the BF was measured for the hamstring muscles group. Finally, although this study focused on the hip extensors and knee flexors, a more global approach appears fundamental for HMI understanding and prevention (Mendiguchia and Brughelli, 2011; Guex and Millet, 2013; Mendiguchia et al., 2017; Buckthorpe et al., 2018; Oakley et al., 2018).

## Conclusion

This study allows better understanding of the determinants of sprint acceleration performance and horizontal force production in fatigue conditions and the role of the hamstring muscles in such conditions, which presents relevant perspectives in practice given the interest of repeated sprint ability in team sports and the importance of HMIs in sports including sprinting and multiple acceleration. The present results show that the muscular determinants of the horizontal force production change in fatigue state: the hamstring muscles seem not to have the same role than in non-fatigue condition. The horizontal force production seems to be more dependent on the function of the hip extensors and *gluteus maximus*. Given the decrease in hamstring muscle strength with fatigue, we can hypothesize that muscle compensatory and kinematics strategies reported in fatigue state could be an adaptation to allow/maintain performance and a protective adaptation to limit hamstring muscle constraints. However, in a case of *gluteus maximus* weakness, this exposes subjects to HMI risk. Although strong conclusions cannot be made, these present results should be used in practice by integrating hip extensors/*gluteus maximus* strengthening in addition to hamstring knee flexors strengthening, as well as endurance-focused training strategies in addition to maximal performance training, and taking into account the win–win performance-prevention strategy: training for performance can help for injury prevention and vice-versa.

## Author Contributions

PE, JM, MB, PS, and J-BM conceived and designed the study. PE, PG, PJ-R, PS, and J-BM performed experimentation and data collection. PE, JL, PA, PG, PS, and J-BM analyzed the data. PE, JM, JL, PJ-R, PS, and J-BM interpreted the results. PE, JM, JL, PS, and J-BM drafted the manuscript and prepared the table and figure. PE, JM, JL, PG, PJ-R, MB, PS, and J-BM edited, critically revised the manuscript, and approved the final version.

## Conflict of Interest Statement

The authors declare that the research was conducted in the absence of any commercial or financial relationships that could be construed as a potential conflict of interest. The handling Editor declared a past co-authorship with one of the authors MB.

## Abbreviations

BF: *biceps femoris*
con: concentric mode of contraction
CV: coefficient of variation
ecc: eccentric mode of contraction
EMG: electromyography
ES: effect size
*F_H_*: horizontal ground force production, contact-averaged horizontal force component
*F_Tot_*: total ground force production, contact-averaged resultant (total) force
*F_v_*: vertical ground force production, contact-averaged vertical force component
Glu: *gluteus maximus*
GRF: ground reaction force
HExt: hip extensors
HFlex: hip flexors
HMI: hamstring muscle injury
ICC: intraclass correlation coefficient
KExt: knee extensors
KFlex: knee flexors
MVIC: maximal voluntary isometric contraction
*P_max_*: maximal power output
PT: peak torque
PTBW: peak torque normalized to body weight
RF: *rectus femoris*
RS: repeated sprints
SD: standard deviation
SEM: standard error of measurement
*SF*: step frequency
*t_a_*: aerial time
*t_c_*: contact time
*t_swing_*: swing time
VL: *vastus lateralis*
*V_max_*: maximal velocity
